# METTL14 decreases FTH1 mRNA stability via m6A methylation to promote sorafenib-induced ferroptosis of cervical cancer

**DOI:** 10.1080/15384047.2024.2349429

**Published:** 2024-05-13

**Authors:** Lijie Li, Jie Zeng, Sili He, Yanfei Yang, Chen Wang

**Affiliations:** a Department of Gynecology and Obstetrics, The Third Xiangya Hospital of Central South University, Changsha, Hunan, P. R. China; b Pharmacy Intravenous Admixture Services, The Third Xiangya Hospital of Central South University, Changsha, Hunan, P. R. China

**Keywords:** Cervical cancer, m6A modification, ferroptosis, METTL14, FTH1

## Abstract

Cervical cancer (CC) is a prevalent malignancy among women worldwide. This study was designed to investigate the role of METTL14 in sorafenib-induced ferroptosis in CC. METTL14 expression and m6A methylation were determined in CC tissues, followed by analyzes correlating these factors with clinical features. Subsequently, METTL14 was knocked down in CC cell lines, and the effects on cell proliferation, mitochondrial morphology and ferroptosis were assessed using CCK-8, microscopy, and markers associated with ferroptosis, respectively. The regulatory relationship between METTL14 and FTH1 was verified using qRT-PCR and luciferase reporter assays. The functional significance of this interaction was further investigated both *in vitro* and *in vivo* by co-transfecting cells with overexpression vectors or shRNAs targeting METTL14 and FTH1 after sorafenib treatment. METTL14 expression and m6A methylation were significantly reduced in CC tissues, and lower METTL14 expression levels were associated with a poorer CC patients’ prognosis. Notably, METTL14 expression increased during sorafenib-induced ferroptosis, and METTL14 knockdown attenuated the ferroptotic response induced by sorafenib in CC cells. FTH1 was identified as a direct target of METTL14, with METTL14 overexpression leading to increased m6A methylation of FTH1 mRNA, resulting in reduced stability and expression of FTH1 in CC. Furthermore, FTH1 overexpression or treatment with LY294002 partially counteracted the promotion of sorafenib-induced ferroptosis by METTL14. *In vivo* xenograft experiments demonstrated that inhibiting METTL14 reduced the anticancer effects of sorafenib, whereas suppression of FTH1 significantly enhanced sorafenib-induced ferroptosis and increased its anticancer efficacy. METTL14 reduces FTH1 mRNA stability through m6A methylation, thereby enhancing sorafenib-induced ferroptosis, which contributes to suppressing CC progression via the PI3K/Akt signaling pathway.

## Introduction

Cervical cancer (CC) is ranked as the fourth most diagnosed cancer and the fourth leading cause of cancer mortality among women.^1^ It is estimated that globally, there will be approximately 4,820,000 new cases and 3,210,000 deaths attributable to CC in the coming years.^2^ In 2020, the World Health Organization identified CC as a significant public health issue and initiated a campaign to eliminate CC, aiming to reduce the incidence to fewer than 4 cases per 100,000 women years.^3^ Despite advancements in medical technology, surgery remains the primary treatment option for early-stage CC, while chemotherapy is commonly used to reduce tumor size before surgical intervention in advanced cases. Sorafenib, a multi-targeted therapy, has been shown to inhibit the progression of CC by blocking the RAF/MEK/ERK signaling pathways.^4^ However, its clinical utility is hampered by low bioavailability and the development of drug resistance.^5^ Thus, understanding the mechanisms underlying resistance to sorafenib is important to enhance its effectiveness in CC treatment.

m6A methylation represents the most prevalent form of mRNA modification, known to influence mRNA stability, localization, and alternative splicing by modulating modifications in the protein-coding sequences (CDS) and untranslated regions (UTR).^6^ Accumulating evidence suggests that m6A modification plays pivotal roles in the pathogenesis of various cancers and contributes to drug resistance, including in CC.^7,8^ Methyltransferase-like 14 (METTL14), a key component of the methyltransferase complex, has been found to be essential for m6A modification. It maintains the integrity of the catalytic complex and facilitates the recognition of RNA substrates.^9^ Recent studies have identified that METTL14 is significantly upregulated in CC^10^ and enhances CC cell proliferation and migration by modulating the m6A levels of the Myc oncogene,^11^ highlighting the involvement of METTL14 in CC development through m6A modification regulation and the need for further investigation to elucidate its precise mechanisms.

Ferroptosis is recognized as an iron-dependent form of cell death, characterized by phospholipid peroxidation driven by reactive oxygen species (ROS), transition metal iron, and phospholipids enriched with polyunsaturated fatty acid chains.^12,13^ Since the term was introduced in 2012, extensive research efforts have aimed to elucidate its underlying mechanisms across various diseases, with numerous studies suggesting that ferroptosis could represent a novel therapeutic strategy for cancer.^14^ Recent findings by Yang et al. have demonstrated that ferroptosis-related genes are significantly correlated with tumor microenvironments and clinical outcomes, indicating their potential utility in prognostication for CC.^15^ It has been shown that Cdc25A enhances ErbB2 expression through the dephosphorylation of PKM2, thereby inhibiting autophagy-mediated ferroptosis in CC.^16^ As a crucial enzyme for m6A modification, METTL14’s activity can be suppressed under hypoxic conditions in a HIF-1α dependent manner, leading to the inhibition of ferroptosis in hepatocellular carcinoma.^17^ In addition, sorafenib, primarily used for the treatment of several cancers, has been associated with the induction of ferroptosis.^18^ Present research indicates that sorafenib can trigger ferroptosis in cancer cells by inhibiting certain cellular mechanisms that protect against oxidative stress, leading to the accumulation of lethal levels of lipid peroxides.^19^ This association is significant because it highlights a potential therapeutic mechanism through which Sorafenib exerts its anti-cancer effects, offering insights into the development of novel cancer treatment strategies that target ferroptosis. However, the specific role of METTL14 in regulating m6A modification and its involvement in sorafenib-induced ferroptosis in CC are yet to be fully clarified.

In this study, we investigated the role of METTL14 in sorafenib-induced ferroptosis in CC. Ferritin heavy chain 1 (FTH1), an important regulator of ferroptosis, was identified as a potential target of METTL14 through bioinformatics analysis and was found to contain numerous m6A modification sites. Thus, we explored the underlying mechanism of the METTL14/FTH1 axis in sorafenib-induced ferroptosis in CC both *in vitro* and *in vivo* to offer new insights into the understanding and treatment of CC.

## Materials and methods

### Clinical samples

A total of 53 pairs of CC samples and corresponding normal adjacent tissues were obtained from the Third Xiangya Hospital of Central South University. All specimens were collected and stored at −80°C for subsequent analysis. The inclusion criteria for participants in this study were as follows: (1) initially diagnosed with CC and had not undergone any preoperative treatments; (2) availability of detailed clinical information, including age, results of imaging examinations, and Federation of Gynecology and Obstetrics (FIGO) stage; (3) availability of at least a 5-year follow-up data; (4) absence of any other cancers or infectious diseases. The exclusion criteria were: (1) loss to follow-up or accidental death; (2) pregnancy, lactation or history of drug addiction; and (3) withdrawal from any other treatment strategies. This study was approved by the Ethics Committee of the Third Xiangya Hospital of Central South University, and informed consent was obtained from all patients.

### Cell culture and treatment

The normal cervical epithelial cell line H8 and cervical cancer cell lines (ME180, HCC9, SiHa, Hela, MS751, and CaSki) were purchased from the National Biomedical Experimental Cell Library (Beijing, China). All cell lines were cultured in Dulbecco’s Modified Eagle’s Medium (DMEM) supplemented with 10% fetal bovine serum and 1% penicillin-streptomycin and were incubated in a humidified atmosphere at 37°C with 5% CO_2_. For drug treatment experiments, cells at 70%-80% confluency were treated with the ferroptosis inducer sorafenib, ferrostatin-1 (Fer-1, 1 μM), or LY294002, either individually or in combination.

### Plasmids and cell transfection

Specific short hairpin RNA (shRNA) constructs targeting METTL14 (sh-METTL14) and FTH1 (sh-FTH1), overexpression plasmids for METTL14 (pcDNA-METTL14) and FTH1 (pcDNA-FTH1), and their corresponding negative controls (sh-NC, pcDNA-NC), were obtained from GenePharma (Shanghai, China). Additionally, sequences representing wild-type METTL14 (WT-METTL14) and mutant METTL14 (MUT-METTL14) were amplified using cDNA as a template and subsequently cloned into the pGL6 vector. The transfection of these plasmids into cells was performed using Lipofectamine 3000 (Invitrogen, Carlsbad, CA, USA) following the manufacturer’s instructions. Cells were harvested 48 hours post-transfection to determine transfection efficiency or for subsequent analyses.

### Cell counting kit-8 (CCK-8) assay

Cell proliferation was assessed using the CCK-8 assay (Beyotime, Shanghai, China), following the manufacturer’s instructions. Briefly, cells subjected to various treatments were plated in 96-well plates and incubated for 24, 48, 72, and 96 h, respectively. Then, CCK-8 solution was added, and the cells were incubated at 37°C for 4 h. Absorbance was measured at 450 nm using a microplate reader (Thermo Fisher Scientific, Waltham, MA, USA).

### Quantification of m6A RNA methylation

The m6A level in total RNA was quantified using the m6A RNA Methylation Assay Kit (ab185912, Abcam) according to the manufacturer’s protocol. Briefly, total RNA was isolated and added to the assay wells. The wells were then washed and incubated with a capture antibody. Following another washing step, the wells were incubated with a detection antibody and enhancer solution as per the provided instructions. After the addition of the color-developing solution, the absorbance at 450 nm for each well was measured using a microplate reader.

### m6A RNA immunoprecipitation PCR (RIP-qPCR)

The m6A methylation level of specific genes was assessed using the MeRIP-qPCR assay, employing the Magna MeRIP m6A Kit (Merck, Germany), following the manufacturer’s instructions. Initially, total RNA was isolated and fragmented by ultrasound. Subsequently, magnetic beads coated with anti-m6A antibody were used to capture the RNA fragments. The m6A-specific salt provided in the kit was utilized to elute the bound RNA, which was then purified using the RNA purification kit (Qiagen, USA). qRT-PCR was performed on the eluted RNA, and the relative levels of m6A modification were calculated using the 2^−ΔΔCt^ method.

### Transmission electron microscope (TEM)

TEM was used to investigate morphological changes in cells. Briefly, samples were collected and centrifuged at 4°C for 5 minutes. The cell pellets were then fixed with 2.5% glutaraldehyde for 2 h at 4°C, and the samples were processed for TEM analysis by Servicebio (Wuhan, China) to examine ultrastructural changes. Based on TEM images, the proportion of shrunken mitochondria was quantified manually in comparison to normal mitochondria.

### Determination of reactive oxygen species (ROS)

The intracellular levels of ROS were measured using the ROS Assay Kit (Beyotime Biotechnology) following the manufacturer’s instructions. Briefly, the cells were incubated with 2ʹ,7ʹ-dichlorofluorescin diacetate (DCFH-DA) in darkness at 37°C for 30 min, allowing the DCFH-DA to permeate the cells and be deacetylated to its fluorescent form by intracellular esterases. Post-incubation, cells were washed three times with phosphate-buffered saline (PBS) to remove excess DCFH-DA and then resuspended in a serum-free medium. Then, the fluorescence intensity was measured using a fluorescence microplate reader (Beckman Coulter, Indianapolis, IN, USA) at wavelength 485 nm.

For lipid ROS determination, BODIPY^TM^ 581/591 C11 (Invitrogen, USA, D3861) was conducted to detect the lipid peroxidation levels. The cells were seeded in 24-well plates and incubated with 10 μM BODIPYTM 581/591 C11 for 0.5 h according to the manufacturer’s operation instruction. Then cells were washed three times with PBS. Fluorescence of different wavelengths was observed and representative images were captured under a fluorescence microscope.

### Detection of glutathione (GSH)

The intracellular GSH level was evaluated using a commercial GSH determination kit (BIOXYTECH GSH-400, Oxis International, Portland, OR, USA) following the provided instructions. Briefly, the cells were seeded in 6-well plates and treated accordingly. After treatment, the cells were scraped and lysed in 5% metaphosphoric acid to precipitate proteins. The lysates were then centrifuged at 12,000 g for 15 min to collect the supernatants. GSH levels were determined by measuring the absorbance at 400 nm using a microplate reader.

### Determination of malondialdehyde (MDA)

Lipid peroxidation levels were measured using a thiobarbituric acid (TBA) reactive substances assay and the MDA assay kit (Nanjing Jincheng Bioengineering Institute) based on the reaction of TBA with MDA under acidic conditions at 95–100°C, resulting in the formation of a pink MDA-TBA adduct. The absorbance of this adduct was measured at 450 nm, and the concentration of MDA was calculated according to the manufacturer’s instructions.

### Determination of Fe^2+^

Intracellular Fe^2+^ ions were detected using the FeRhoNox™-1 fluorescent probe (GORYO Chemical, Sapporo, Japan). Briefly, the cells were seeded on coverslips and subjected to different treatments. Then, the medium was discarded, rinsed with phosphate-buffered saline (PBS) twice, and incubated with 5 μM FeRhoNox™-1 solution in the dark at 37°C for 1 h. After rinsing with PBS three times, the cells were analyzed using a Nikon A1Si Laser Scanning Confocal Microscope (Nikon Instruments Inc.).

### RNA stability detection

After treatment with 5 μg/ml Actinomycin D, the cells were incubated for 0, 3, and 6 hours, after which they were harvested for RNA extraction using TRIzol reagent as described previously.^20^ Subsequently, the rate of mRNA degradation was determined based on methods outlined in the published literature.

### Dual luciferase activity reporter assay

The interaction between METTL14 and FTH1 was assessed via a luciferase reporter assay. The cells were co-transfected with either wild-type or mutant FTH1 plasmids, the PGL6-METTL14 luciferase reporter plasmid, and the pRL-TK Renilla luciferase reporter vectors. Forty-eight hours post-transfection, both firefly and Renilla luciferase activities were measured using the Dual-Luciferase Reporter Assay System (Promega), following the manufacturer’s instructions.

### Quantitative real-time polymerase chain reaction (qRT-PCR)

Total RNA from cervical tissue samples and cell lines was extracted using TRIzol reagent (Takara, Dalian, China), following the manufacturer’s protocol, and its quality was assessed using a Nanodrop 2000 spectrophotometer (Thermo). Subsequently, 2 μg of total RNA was used for cDNA synthesis employing M-MLV Reverse Transcriptase (Takara), according to the provided instructions. qRT-PCR was performed using SYBR Green (Thermo) on an ABI 6500 system (Applied Biosystems, USA) under the following conditions: initial denaturation at 95°C for 15 min, followed by 40 cycles of denaturation at 95°C for 10 s, annealing at 58°C for 25 s, and extension at 72°C for 15 s. β-actin was used as the internal control, and the relative expression levels of specific genes were calculated using the 2^−ΔΔCt^ method. The primers used for qRT-PCR are listed in Table 1.

**Table 1.** Primer sequences for qRT-PCR.

Gene	Forward (5’-3’)	Reverse (5’-3’)
METTL14	AGAAACTTGCAGGGCTTCCT	TCTTCTTCATATGGCAAATTTTCTT
FTH1	ACATCAAGAAGGTGGTGAAGC	AAGGTGGAAGAGTGGGAGTTG
GAPDH	ACATCAAGAAGGTGGTGAAGC	AAGGTGGAAGAGTGGGAGTTG

### Western blot

Tissue or cell samples were lysed using RIPA lysis buffer (Beyotime, Shanghai, China) supplemented with 1% PMSF and 0.1% protease inhibitor cocktail and incubated on ice for 10 min. The lysates were then centrifuged, and the supernatant was collected for protein quantification using a BCA Protein Assay Kit (Beyotime, China). Subsequently, 30 μg of protein per sample was separated by SDS-PAGE and transferred onto a PVDF membrane (Pierce, Rockford, IL, USA). The membrane was blocked with 5% nonfat milk at room temperature for 1 h and incubated overnight at 4°C with the following primary antibodies: anti-METTL14 (1:1000 dilution; #ab300104, Abcam, Cambridge, MA, USA), anti-FTH1 (1:3000 dilution; #ab65080, Abcam), anti-GPX4 (1:2000 dilution; #ab125066, Abcam), anti-SLC7A11 (1:10000 dilution; #ab175186, Abcam), anti-PI3K (1:1000 dilution; #ab283852, Abcam), anti-p-PI3K (0.5 μg/ml dilution; #ab278545, Abcam), anti-Akt (1:1000 dilution; #ab38449, Abcam), anti-p-Akt (1:5000 dilution; #ab81283, Abcam), and anti-β-actin (1:2000 dilution; #ab8227, Abcam). Following incubation with the indicated primary antibodies, the membrane was incubated with a horseradish peroxidase-conjugated anti-rabbit secondary antibody (1:5000 dilution; #ab99697, Abcam) at room temperature for 1 h. Protein bands were detected using an enhanced chemiluminescence (ECL) method (Pierce) and quantified with ImageJ software version 1.52 (National Institutes of Health, Bethesda, MD, USA).

### Animal studies

For the tumor xenograft experiments, BALB/c athymic female nude mice (4 weeks old, obtained from Beijing Vital River Laboratory Animal Technology Co., Ltd) were utilized. The mice were randomly allocated into five groups, each consisting of 5 mice. For xenograft establishment, HCC9 cells, transfected with sh-METTL14, sh-FTH1, or both, were harvested, and 5 × 10^5^ HCC9 stable transfected cells were subcutaneously injected into the right flank of each mouse. Tumor volume was measured weekly using a vernier caliper and calculated with the formula: Volume = (length × width^2^/2, where length is the longest diameter of the tumor, and width is the diameter perpendicular to the length. Seven weeks post-inoculation, all mice were euthanized, and the tumors were excised, weighed, and processed for further analyses.

### Hematoxylin and eosin (H&E) staining

Tissue morphology assessment was performed using H&E staining. Briefly, the tumor tissues were fixed in 4% paraformaldehyde, embedded in paraffin, and sectioned into 5-μm slices. Subsequently, the sections underwent H&E staining following the manufacturer’s protocol (Nanjing Jiancheng, Nanjing, Jiangsu, China).

### Statistical analyses

In this study, all the experiments were conducted thrice, and data are presented as the mean ± standard deviation (SD) of the obtained results. Statistical analyses were performed using GraphPad Prism version 7.0 (GraphPad Software Inc., San Diego, CA, USA). Differences between two groups were assessed using the unpaired Student’s t-test or the chi-square test, as appropriate. Comparisons between multiple groups were conducted using one-way analysis of variance (ANOVA) followed by Duncan’s post hoc test for specific pairwise comparisons to determine specific pairwise differences at each of the indicated time points. The Kaplan-Meier method was employed to analyze the survival rates of patients categorized based on high versus low expression levels of METTL14. The correlation between FTH1 and METTL14 expression was evaluated using Pearson correlation analysis. A P-value of < 0.05 was considered statistically significant.

## Results

### Downregulated METTL14 is associated with poor prognosis in CC patients

**Figure 1.**
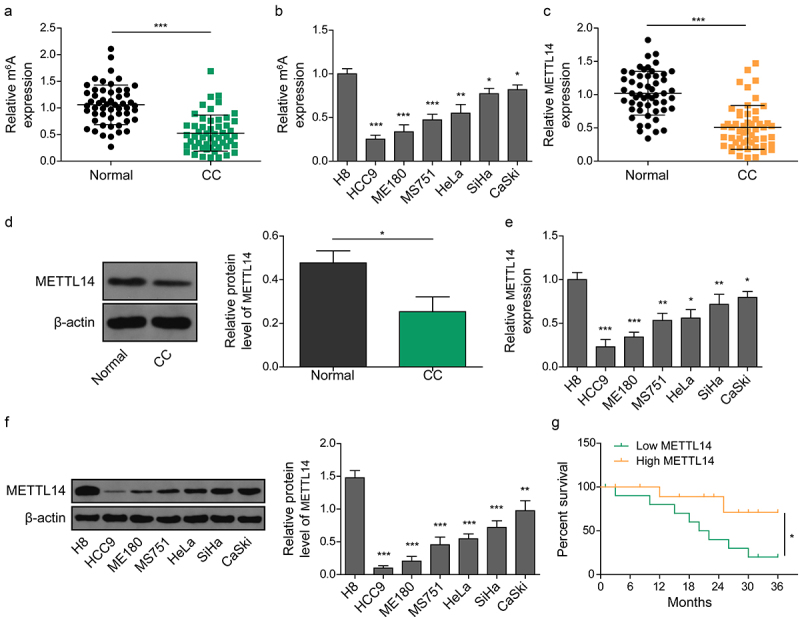
METTL14 is downregulated in CC tissues and correlates with poor prognosis of CC patients. (a,b) determination of the m6A content in CC tissue (A) and cell lines (B). (c,d) METTL14 expression in CC tissues was determined by qRT-PCR (C) and western blotting (D). (e,f) expression of METTL14 in CC cell lines determined by qRT-PCR (E) and western blotting (F). (g) The survival outcome of CC patients with low and high expression of METTL14. *, *p* < .05; **, *p* < .01; and ***, *p* < .001.

Clinical sample analysis revealed a significantly reduced level of m6A modification in CC tissues compared to non-tumor tissues (Figure 1(a)). Furthermore, analysis of cell lines showed that m6A modification levels in CC cell lines (HCC9, ME180, MS751, Hela, SiHa, and CaSki) were significantly lower than those in the normal cervical epithelial cell line H8, particularly between H8 and HCC9 cells (Figure 1(b)), indicating a potential role of decreased m6A modification in CC progression. METTL14, an important enzyme for m6A modification, was assessed in CC tissues and cell lines through qRT-PCR and Western blotting assays. The results demonstrated a significant reduction of METTL14 in CC tissues and cell lines compared to their respective controls (Figure 1(c–f)). Additionally, the correlation between METTL14 expression and clinical-pathological characteristics in CC patients was examined. Fifty-three CC patients were divided into two groups (Low-METTL14 group and High-METTL14 group) based on the median expression level of METTL14. As shown in Table 2, METTL14 expression in CC tissues was significantly correlated with tumor size, differentiation, lymph node metastasis, and FIGO stages. Kaplan-Meier survival analysis further indicated that lower expression of METTL14 was significantly associated with shorter overall survival times compared to higher expression (Figure 1(g)). Taken together, these observations suggest that METTL14 is significantly decreased in CC and is linked to poorer prognosis through its role in m6A modification.

**Table 2.** Correlation between METTL14 expression and clinical pathological characteristics in cervical cancer patients (*n* = 53).

Characteristics	Number	METTL14		*p*-value
		Low (*n* = 27)	High (*n* = 26)	
**Age**				0.678
< 45	27	13	14	
≥45	26	14	12	
**Histomorphology**				0.410
Squamous cell carcinoma	20	8	12	
Adenocarcinoma	22	12	10	
Adenosquamous carcinoma	11	7	4	
**Tumor size (cm)**				0.074*
< 4	26	10	16	
≥4	27	17	10	
**Lymph-node metastasis**				0.009*
Negative	25	8	17	
Positive	28	19	9	
**Distal metastasis**				0.132
Negative	25	10	15	
Positive	28	17	11	
**Differentiation**				0.038*
Low	20	8	12	
Medium	16	6	10	
High	17	13	4	
**FIGO stages**				
I	19	5	14	0.027*
II	26	17	9	
III – IV	8	5	3	
**HPV**				0.252
HPV16+	22	9	13	
HPV18+	23	12	11	
Others	8	6	2	

The median of relative METTL14 expression level is 0.48, so the number of low METTL14 expression is 26 (<0.48). FIGO: International Federation of Gynecology and Obstetrics. * *p* < .05.

### METTL14 is upregulated during sorafenib-induced ferroptosis in CC cells

**Figure 2.**
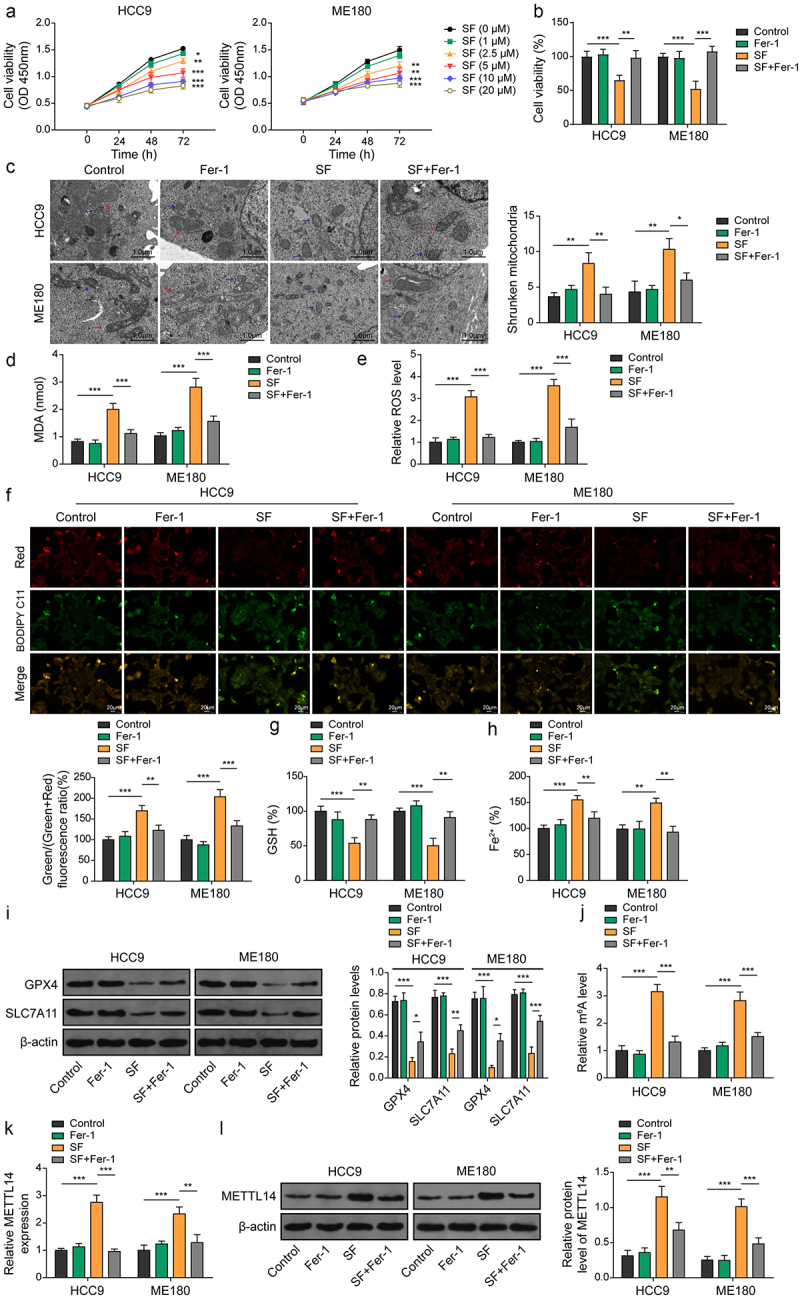
METTL14 is upregulated during sorafenib-induced ferroptosis. (a) Cell survival ability of CC cells after pre-treatment with different concentrations (0, 1, 2.5, 5, 10, 20 μM) of sorafenib for 48 h, measured at the indicated time and the results were compared with the control group. (B-L) CC cells were pre-treated with sorafenib (10 μM, 48 h) and/or administration of ferroptosis inhibitor fer-1. (b) Cell survival ability of CC cells was measured using CCK-8. (c) Mitochondrial morphology alterations in cells were determined using TEM. Red arrows: typical mitochondria morphology. Blue arrows: shrunken mitochondria morphology. (scale bar = 1 μm). (d–h) MDA (D), ROS (E), lipid peroxidation (F), GSH (G) and Fe^2+^ (H) levels in cells were evaluated using commercial kits. (i) Protein levels of GPX4 and SLC7A11 were verified by western blotting. (j) m6A levels were determined using RIP-qPCR. (k,l) expression of METTL14 in cells was determined by qRT-PCR (J) and western blotting (K). *, *p* < .05; **, *p* < .01; and ***, *p* < .001.

Based on the analysis of m6A and METTL14 expression, HCC9 and ME180, the cell lines with the most significant downregulation, were selected for further studies. Initially, HCC9 and ME180 cells were exposed to various concentrations of sorafenib to determine an optimal dose for treatment. The findings indicated that 10 μM sorafenib significantly inhibited the viability of CC cells without a notable difference compared to the 20 μM concentration (Figure 2(a)). Thus, 10 μM sorafenib for 48 h was selected for subsequent experiments. To investigate the potential involvement of ferroptosis, HCC9 and ME180 cells were treated with sorafenib alone or in combination with Fer-1, followed by cell viability and morphology assessments. The CCK-8 assay results revealed that Fer-1 significantly counteracted the decrease in viability of HCC9 and ME180 cells induced by sorafenib (Figure 2(b)). TEM analysis showed that sorafenib treatment led to a significant increase in the proportion of cells with shrunken mitochondria, characterized by reduced volume, denser membranes, and diminished mitochondrial cristae. In contrast, Fer-1 treatment effectively mitigated these alterations, suggesting that sorafenib may induce ferroptosis in CC cells (Figure 2(c)). Further biochemical analyses demonstrated that sorafenib treatment significantly elevated levels of MDA, ROS, lipid peroxidation and Fe^2+^, while reducing GSH levels in HCC9 and ME180 cells. Fer-1 alone had no impact on these parameters but significantly attenuated the changes induced by sorafenib in HCC9 and ME180 cells (Figure 2(d–g)). Western blot analysis revealed that Fer-1 treatment alone did not affect m6A modification or METTL14 expression but significantly reversed the upregulation of m6A and METTL14 expression induced by ferroptosis in HCC9 and ME180 cells (Figure 2(h–l)). These results suggest that sorafenib induces ferroptosis and is associated with an increase in METTL14 expression in CC cell lines.

### Silencing METTL14 inhibits sorafenib-induced ferroptosis in CC cells

**Figure 3.**
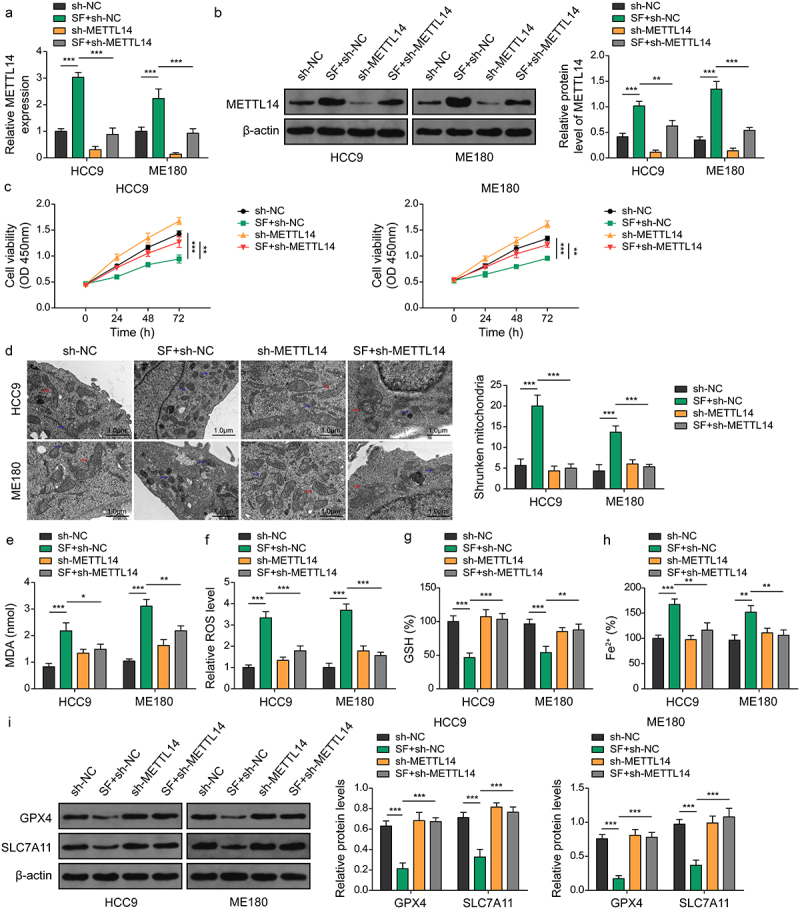
Silencing of METTL14 inhibits sorafenib-induced ferroptosis in CC cells. (a–i) CC cell lines were transfected with sh-NC or sh-METTL14 and treated with sorafenib. (A-B) expression of METTL14 in cells was determined by qRT-PCR (A) and western blotting (B). (C) Cell viability was verified using CCk-8 assays. (D) Mitochondrial morphology alterations in cells were evaluated by TEM. Red arrows: typical mitochondria morphology. Blue arrows: shrunken mitochondria morphology. (scale bar = 1 μm). (E-H) MDA (D), ROS (E), GSH (F) and Fe^2+^ (G) levels in cells were determined using commercial kits. (I) Protein levels of GPX4 and SLC7A11 were confirmed by western blotting. *, *p* < .05; **, *p* < .01; and ***, *p* < .001.

To further explore the function of METTL14 in sorafenib-exposed CC cells, METTL14 expression was knocked down in HCC9 and ME180 cells, followed by cell proliferation and ferroptosis-related markers assessments. Both qRT-PCR and western blot analyses confirmed the effective downregulation of METTL14 in these cell lines. Interestingly, sorafenib treatment was observed to partially counteract the suppression of METTL14 (Figure 3(a,b)). Silencing METTL14 significantly promoted the proliferation of HCC9 and ME180 cells compared to control, while the increased proliferation observed upon METTL14 knockdown was mitigated by sorafenib treatment (Figure 3(c)). Mitochondrial morphology analysis revealed that METTL14 downregulation partially abolished the increase in shrunken mitochondria induced by sorafenib (Figure 3(d)). Biochemical assays further demonstrated that METTL14 inhibition partially reversed the sorafenib-induced elevation of MDA, ROS and Fe^2+^ levels, as well as a reduction in GSH levels in HCC9 and ME180 cells (Figure 3(e–h)). Moreover, western blot analysis showed that silencing METTL14 significantly mitigated the decrease in the expression of glutathione peroxidase 4 (GPX4) and SLC7A11, key markers of ferroptosis, in cells treated with sorafenib (Figure 3(i)). These findings suggest that the inhibition of METTL14 can counteract the ferroptosis induced by sorafenib in CC cells, highlighting the pivotal role of METTL14 in mediating the cellular response to sorafenib treatment.

### METTL14 destabilizes FTH1 mRNA via m6A modification

**Figure 4.**
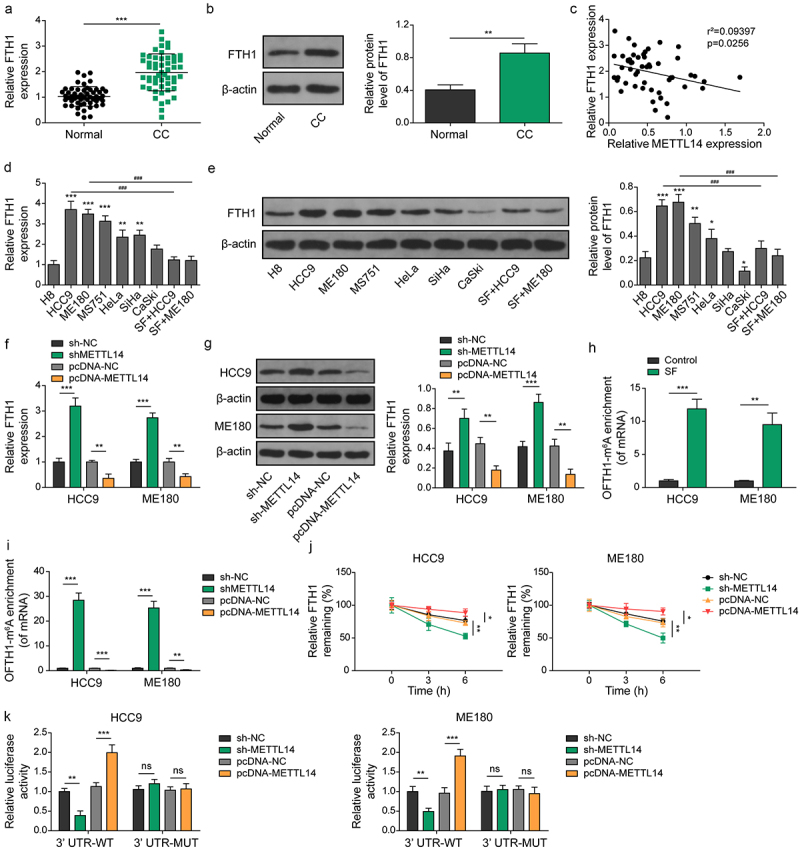
METTL14 decreases the stability of FTH1 in an m6A-dependent manner. (a,b) the mRNA expression of FTH1 in CC was determined using qRT-PCR (A) and western blotting (B). (C) Correlation between FTH1 and METTL14 was assessed using Pearson analysis. (d,e) the mRNA expression of FTH1 in different CC cell lines was detected using qRT-PCR (D) and western blotting (E). Of note, *, ** or *** indicates results compared to the H8 cell, while ### indicates results compared to HCC9 or ME180. (f,g) expression of FTH1 in CC cell lines after transfecting with pcDNA-METTL14 or sh-METTL14 were confirmed by qRT-PCR (F) and western blotting (G). (h,i) the m6A methylation level of FTH1 mRNA in CC cell lines after treating with sorafenib (H) or transfecting with pcDNA-METTL14 or sh-METTL14 (I) determined using RIP-qPCR. (J) Detection of FTH1 mRNA stability by qRT-PCR in CC cell lines after transfecting with pcDNA-METTL14 or sh-METTL14. (K) Regulation between METTL14 and FTH1 was verified by luciferase activity reporter assay. Of note, no significant differences were observed among the groups with MUT 3’UTR. *, *p* < .05; *p* < .01; ***, *p* < .001; and ###, *p* < .001.

FTH1, a subunit of ferritin, plays a significant role in iron homeostasis. Analysis of clinical samples revealed a significant upregulation of FTH1 in CC (Figure 4(a,b)), which was closely correlated with METTL14 expression (Figure 4(c)). Notably, HCC9 and ME180 cells exhibited the highest levels of FTH1 expression compared to H8 cells, and sorafenib treatment resulted in a significant reduction of FTH1 expression in these cell lines (Figure 4(d,e)). Further investigation indicated that upregulation of METTL14 led to a decrease in FTH1 expression, whereas METTL14 silencing significantly increased FTH1 levels in HCC9 and ME180 cells (Figure 4(f,g)). Based on these observations, it was hypothesized that METTL14 could influence FTH1 expression through m6A modification. RIP-qPCR analysis revealed that sorafenib treatment and METTL14 overexpression significantly increased m6A modification of FTH1 mRNA. Conversely, inhibition of METTL14 resulted in a significant decrease in m6A modification of FTH1, suggesting that METTL14 regulates the m6A modification of FTH1 (Figure 4(h,i)). To assess the impact of METTL14 on FTH1 mRNA stability, it was found that METTL14 overexpression significantly reduced FTH1 mRNA stability, while METTL14 inhibition notably increased its stability (Figure 4(j)). Additionally, luciferase activity reporter assay demonstrated that METTL14 overexpression significantly reduced the activity of the wild-type 3′-UTR of FTH1 mRNA, but did not affect the mutant 3′-UTR activity (Figure 4(k)). These findings indicate that METTL14 reduces FTH1 expression by promoting m6A modification-mediated mRNA degradation.

### METTL14 promotes sorafenib induced ferroptosis via FTH1 mediated PI3K/Akt signaling pathway

**Figure 5.**
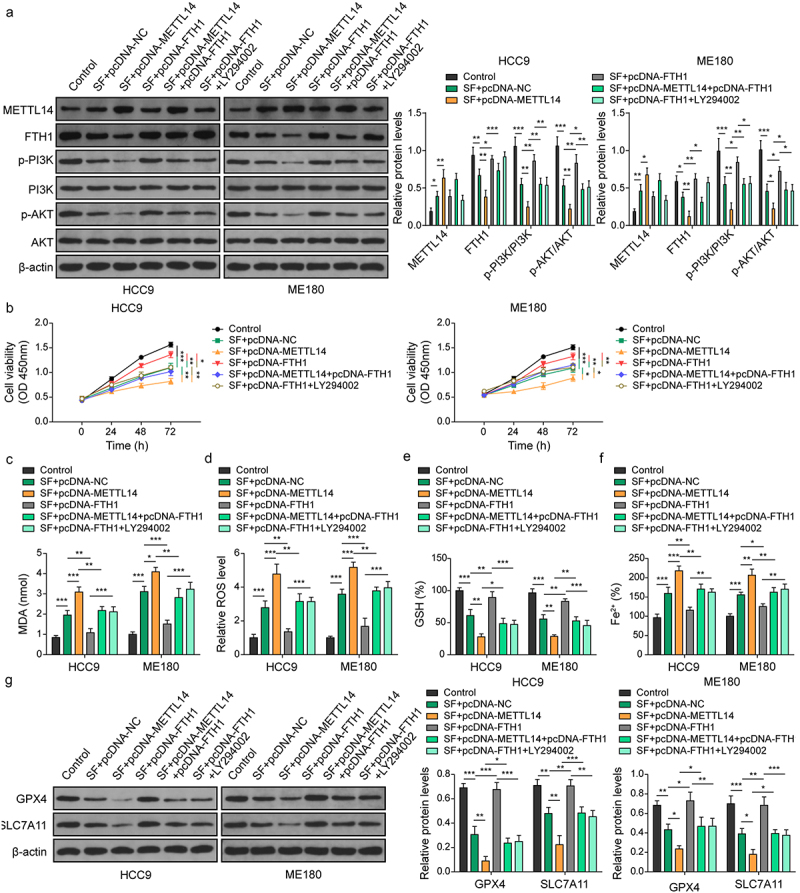
METTL14 promotes sorafenib-induced ferroptosis via FTH1 mediated PI3K/Akt pathway. CC cell lines were transfected with pcDNA-METTL14 and/or pcDNA-FTH1 and treated by sorafenib treatment. (a) Western blotting was conducted to detect protein levels of METTL14, FTH1, PI3K, AKT, and phosphorylation of PI3K and akt. (b) Cell viability was confirmed by CCK-8 following treatment with 10 μM sorafenib for 48 h. (c–f) levels of MDA (C), ROS (D), GSH (E) and Fe^2+^ (F) levels in cells were assessed by commercial kits. (G) Expression of GPX4 and SLC7A11 was determined using western blotting. *, *p* < .05; **, *p* < .01; and ***, *p* < .001.

Previous studies have demonstrated that the PI3K/Akt signaling pathway is commonly aberrantly activated in various tumors, such as hepatocellular carcinoma^21^ and colorectal cancer.^22^ Evidence indicated that inhibiting the PI3K/Akt pathway may sensitize cancer cells to ferroptosis induction, offering a promising strategy to prevent carcinogenic progression.^23^ Therefore, for further exploration of the underlying signaling pathway, we investigated the effects of METTL14 and FTH1 on the PI3K/Akt pathway. As illuminated in Figure 5(a), the results from Western blot assay demonstrated that overexpression of METTL14 further increased the upregulation of METTL14 expression induced by SF, and further inhibited the expression of FTH1 suppressed by sorafenib in HCC9 and ME180 cell lines. Besides, overexpression of FTH1 greatly restored the suppressed expression of FTH1 by SF and reversed the inhibitory effect of METTL14 overexpression on FTH1. Moreover, the results suggested that overexpression of METTL14 intensified the sorafenib-induced reduction of p-PI3K and p-Akt, whereas overexpression of FTH1 or treatment with LY294002, a PI3K inhibitor, significantly counteracted the impact of METTL14 on PI3K and Akt phosphorylation (Figure 5(a)). Cell proliferation assays revealed that METTL14 overexpression significantly increased the anti-proliferative effect of sorafenib on HCC9 and ME180 cells. Conversely, FTH1 overexpression or LY294002 treatment significantly reduced the inhibitory influence of METTL14 on cell proliferation (Figure 5(b)). Biochemical analyses confirmed that METTL14 overexpression significantly intensified the sorafenib-induced alterations in HCC9 and ME180 cells, including increased levels of MDA, ROS and Fe^2+^, and decreased GSH levels. However, these effects were partially reversed by FTH1 overexpression or LY294002 treatment (Figure 5(c–h)). Western blot analysis further revealed that METTL14 overexpression significantly augmented the sorafenib-induced decrease in GPX4 and SLC7A11 expression in HCC9 and ME180 cells, effects which were reversed by FTH1 overexpression or LY294002 treatment (Figure 5(g)). These results suggest that METTL14 enhances the anti-proliferative effects of sorafenib by modulating the PI3K/Akt pathway, while FTH1 and PI3K inhibition counteract this modulation, impacting cell proliferation and survival dynamics in cancer cells.

### METTL14 promoted sorafenib resistance via enhancing FTH1-mediated ferroptosis

**Figure 6.**
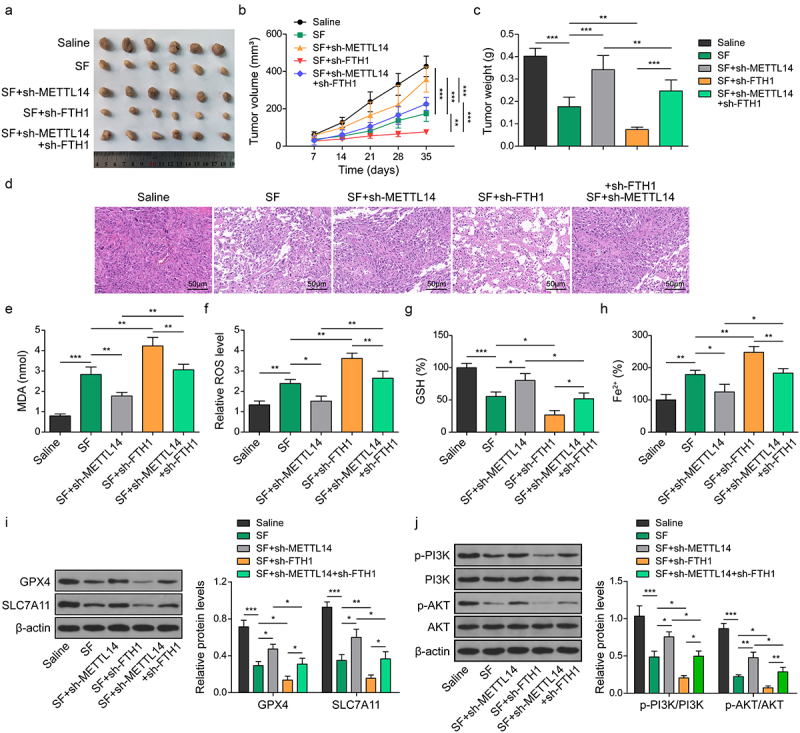
METTL14 enhances the sorafenib sensitivity of CC cells via inhibiting FTH1.

Next, we validated our findings *in vivo* using a xenograft model. The tumor growth curve and volume measurements revealed that sorafenib significantly curtailed tumor growth. Silencing METTL14 mitigated the inhibitory effects of sorafenib on tumor growth, whereas silencing FTH1 significantly increased sorafenib’s efficacy in suppressing tumor growth, thereby counteracting the effects of METTL14 silencing (Figure 6(a–c)). Histological examination indicated that METTL14 inhibition notably reduced sorafenib-induced cellular necrosis, while silencing FTH1 considerably amplified the effects of sorafenib and counteracted the resistance induced by METTL14 silencing (Figure 6(d)). Furthermore, silencing METTL14 significantly diminished the sorafenib-induced increase in MDA, ROS and Fe^2+^ levels and the decrease in GSH, GPX4 and SLC7A11 levels. In contrast, FTH1 suppression significantly reversed the effects of METTL14 silencing and enhanced the sorafenib-induced alterations (Figure 6(e–j)). These findings indicate that METTL14 contributes to sorafenib resistance in CC by inhibiting FTH1 and enhancing downstream ferroptosis.

(a–h) HCC9 cells transfected with sh-METTL14 or/and sh-FTH1 were assessed in a xenograft model using nude mice and treated with sorafenib. (A-C) Representative images of tumors from each group mice (A). Tumor volume growth curve (B), tumor weight (C) of each group mice. (D) H&E staining of the tumor tissues. (E-H) Levels of MDA (E), ROS (F), GSH (G) and Fe^2+^ (H) levels in cells were determined using commercial kits. (i) Protein levels of GPX4 and SLC7A11 were assessed using western blotting. (j) Expression and phosphorylation of PI3K and Akt in tumor tissues were determined using western blotting. *, *p* < .05; **, *p* < .01; and ***, *p* < .001.

## Discussion

CC ranks as the fourth most prevalent cancer among women, with approximately 90% of cases occurring in low- and middle-income countries.^24^ Despite the widespread use of sorafenib as a chemotherapeutic agent, resistance to it remains a major hurdle in treatment. Herein, our study identifies METTL14 as significantly underexpressed in CC tissues but could be upregulated by sorafenib-induced ferroptosis in cell lines. We discovered that METTL14 targets FTH1, inhibiting its expression through enhanced m6A mRNA modification, thereby modulating sorafenib resistance. This mechanism underscores the potential of the METTL14-FTH1 pathway as a therapeutic target to counteract sorafenib resistance in CC. By investigating the molecular interplay between METTL14 and ferroptosis via FTH1 regulation, our findings pave the way for potential novel strategies to enhance sorafenib efficacy, aiming to improve treatment outcomes for CC patients and address a critical public health challenge.

METTL14, an m6A methyltransferase, catalyzes RNA m6A modification by forming a complex with METTL3 and WTAP.^25^ Recent research demonstrated that piRNA-14633 significantly increases METTL14 mRNA stability, thereby enhancing m6A RNA methylation in CC.^26^ Furthermore, METTL14 has been implicated in correlating with the infiltration of tumor-associated macrophages and the prognosis of CC. The lactate produced by tumor glycolysis, serving as a proinflammatory and immunosuppressive mediator within the tumor microenvironment, is modulated by METTL14-mediated m6A methylation, influencing the polarization of macrophages.^27^ Our study identifies that METTL14 and m6A modification are significantly downregulated in CC and that there is a correlation between METTL14 levels and poor prognosis in CC. Another study highlighted that hypoxia suppresses METTL14 in a HIF-1α-dependent manner and that METTL14 mediates m6A modification of SLC7A11 mRNA at its 5′-UTR, leading to a decrease in SLC7A11 expression via a YTHDF2-dependent pathway, thus inhibiting ferroptosis in hepatocellular carcinoma.^17^ In our investigations, we also explored ferroptosis and discovered that METTL14 is upregulated in sorafenib-induced ferroptosis while silencing METTL14 significantly inhibits sorafenib-induced ferroptosis in CC. These observations suggest that METTL14 may be positively associated with sorafenib resistance in CC through its mediation of m6A modification.

Ferritin, a protein complex, comprises two chains: the light chain (FTL) at 19 KDa and the heavy chain (FTH1) at 21 KDa.^28^ FTH1 functions as a ferroxidase, catalyzing the oxidation of ferrous iron to ferric iron.^29^ Our findings revealed a significant upregulation of FTH1 in CC and found it to be inversely correlated with METTL14 expression. Additionally, sorafenib treatment was observed to significantly decrease the m6A level of FTH1 mRNA, while METTL14 overexpression increased FTH1 mRNA m6A modification but reduced FTH1 expression. Zhu et al. reported that DARS-AS1 is significantly upregulated in CC tissues and facilitates DARS translation through recruitment of Mettl3 and METTL14-mediated m6A methylation, thus regulating hypoxia-induced cytoprotective autophagy in CC.^30^ Based on these findings, we hypothesize that METTL14 may promote sorafenib-induced ferroptosis by enhancing FTH1 m6A modification and reducing FTH1 expression, thereby inhibiting CC progression. Previous research has shown that FTH1 is an important regulator in baicalin-induced ferroptosis, with increased FTH1 overexpression mitigating baicalin’s anticancer effects in bladder cancer.^31^ FTH1 also inhibits ferroptosis via ferritinophagy in 6-hydroxydopamine-induced Parkinson’s disease.^32^ Furthermore, curcumenol was found to impede the progression of lung cancer through the lncRNA H19/miR-19b-3p/FTH1 axis-mediated ferroptosis.^33^ As a suppressor of ferroptosis, FTH1 has also been reported to correlate with the polarization and infiltration of macrophages and poor prognosis in head and neck squamous cell carcinoma.^34^ In our study, FTH1 significantly reduced MDA, ROS and Fe^2+^ levels while increasing GSH, GPX4 and SLC7A11 levels in sorafenib-treated CC cells, while METTL14 overexpression or PI3K/Akt inhibitor LY294002 effectively reversed these effects. These observations suggest that METTL14 could enhance sorafenib-induced ferroptosis in CC via the FTH1/PI3K/Akt signaling pathway. Furthermore, xenograft analyses indicate that targeting METTL14 significantly promotes sorafenib sensitivity in CC by enhancing ferroptosis through the inhibition of FTH1 expression.

While providing valuable insights into sorafenib resistance in CC, this study had some limitations. Firstly, although we have explored the correlations between METTL14 expression and clinical features of CC, the impact of varying METTL14 levels on patient prognosis and response to sorafenib treatment necessitates further validation with a larger cohort in future studies. Secondly, while the regulatory connection between METTL14 and FTH1 has been established, the precise role of FTH1 in modulating disease progression and resistance to sorafenib requires more detailed exploration in subsequent research. Despite these limitations, our findings offer novel insights into the mechanisms underlying sorafenib resistance in CC, laying a foundation for future and more comprehensive investigations.

## Conclusion

In conclusion, this study demonstrates that METTL14 is significantly downregulated in CC and that silencing METTL14 markedly reduces sorafenib-induced ferroptosis. In addition, METTL14 suppresses FTH1 expression by promoting m6A modification-mediated mRNA degradation and enhances sorafenib-induced ferroptosis through the FTH1-mediated PI3K/Akt signaling pathway, contributing to the inhibition of CC progression. Therefore, targeting METTL14 and/or FTH1 could be a promising strategy for overcoming sorafenib resistance in CC, although further research is needed to elucidate the detailed mechanism and validate the effectiveness of such approaches.

## Data Availability

All data generated or analyzed during this study are included in this published article.

## References

[1] Sung H, Ferlay J, Siegel RL, Laversanne M, Soerjomataram I, Jemal A, Bray F. Global cancer statistics 2020: GLOBOCAN estimates of incidence and mortality worldwide for 36 cancers in 185 countries. CA Cancer J Clin. 2021;71(3):209–14. doi:10.3322/caac.21660. 33538338

[2] Xia C, Dong X, Li H, Cao M, Sun D, He S, Yang F, Yan X, Zhang S, Li N, et al. Cancer statistics in China and United States, 2022: profiles, trends, and determinants. Chin Med J. 2022; 135(5):584–590. doi:10.1097/CM9.0000000000002108. 35143424 PMC8920425

[3] Bruni L, Serrano B, Roura E, Alemany L, Cowan M, Herrero R, Poljak M, Murillo R, Broutet N, Riley LM, et al. Cervical cancer screening programmes and age-specific coverage estimates for 202 countries and territories worldwide: a review and synthetic analysis. Lancet Global Health. 2022; 10(8):e1115–e27. doi:10.1016/S2214-109X(22)00241-8. 35839811 PMC9296658

[4] Rizzuto I, Otter SJ, Bharathan R, Stewart A. Vascular endothelial growth factor (VEGF) inhibitors for the treatment of metastatic and recurrent cervical cancer. Cochrane Database Syst Rev. 2020. 10.1002/14651858.CD013605. PMC8428759 33661538

[5] Wang J, Lv F, Sun T, Zhao S, Chen H, Liu Y, Liu Z. Sorafenib nanomicelles effectively shrink tumors by vaginal administration for preoperative chemotherapy of cervical cancer. Nanomaterials. 2021;11(12):3271. doi:10.3390/nano11123271. 34947619 PMC8705954

[6] Lan Q, Liu PY, Haase J, Bell JL, Hüttelmaier S, Liu T. The critical role of RNA m6A methylation in cancer. Cancer Res. 2019;79(7):1285–1292. doi:10.1158/0008-5472.CAN-18-2965. 30894375

[7] Paramasivam A, Priyadharsini JV. RNA N6-methyladenosine: a new player in autophagy-mediated anti-cancer drug resistance. Br J Cancer. 2021;124(10):1621–1622. doi:10.1038/s41416-021-01314-z. 33723389 PMC8110764

[8] Ma X, Li Y, Wen J, Zhao Y. m6A RNA methylation regulators contribute to malignant development and have a clinical prognostic effect on cervical cancer. Am J Transl Res. 2020;12(12):8137–8146. 33437387 PMC7791487

[9] Zhou H, Yin K, Zhang Y, Tian J, Wang S. The RNA m6A writer METTL14 in cancers: roles, structures, and applications. Biochimica Et Biophysica Acta (BBA) - Rev Cancer. 2021;1876(2):188609. doi:10.1016/j.bbcan.2021.188609. 34375716

[10] Geng F, Fan M-J, Li J, Liang S-M, C-Y L, Li N. Knockdown of METTL14 inhibits the growth and invasion of cervical cancer. Transl Cancer Res. 2019;8(6):2307. doi:10.21037/tcr.2019.09.48. 35116983 PMC8797423

[11] Hu C, Liu T, Xu Y, Han C, Yang S, Yang K. METTL14 promotes the proliferation and migration of cervical cancer cells by up-regulating m(6)A myc expression. Xi Bao Yu Fen Zi Mian Yi Xue Za Zhi. 2022;38(2):131–137. 35356881

[12] Jiang X, Stockwell BR, Conrad M. Ferroptosis: mechanisms, biology and role in disease. Nat Rev Mol Cell Biol. 2021;22(4):266–282. doi:10.1038/s41580-020-00324-8. 33495651 PMC8142022

[13] Tang D, Chen X, Kang R, Kroemer G. Ferroptosis: molecular mechanisms and health implications. Cell Res. 2021;31(2):107–125. doi:10.1038/s41422-020-00441-1. 33268902 PMC8026611

[14] Shen Z, Song J, Yung BC, Zhou Z, Wu A, Chen X. Emerging strategies of cancer therapy based on ferroptosis. Adv Mater. 2018;30(12):1704007. doi:10.1002/adma.201704007. PMC6377162 29356212

[15] Yang X, Yin F, Liu Q, Ma Y, Zhang H, Guo P, Wen W, Guo X, Wu Y, Yang Z, et al. Ferroptosis-related genes identify tumor immune microenvironment characterization for the prediction of prognosis in cervical cancer. Ann Transl Med. 2022; 10(2):123. doi:10.21037/atm-21-6265. 35282071 PMC8848400

[16] Wang C, Zeng J, L-J L, Xue M, S-L H. Cdc25A inhibits autophagy-mediated ferroptosis by upregulating ErbB2 through PKM2 dephosphorylation in cervical cancer cells. Cell Death Disease. 2021;12(11):1055. doi:10.1038/s41419-021-04342-y. 34743185 PMC8572225

[17] Fan Z, Yang G, Zhang W, Liu Q, Liu G, Liu P, Xu L, Wang J, Yan Z, Han H, et al. Hypoxia blocks ferroptosis of hepatocellular carcinoma via suppression of METTL14 triggered YTHDF2-dependent silencing of SLC7A11. J Cell Mol Med. 2021; 25(21):10197–10212. doi:10.1111/jcmm.16957. 34609072 PMC8572766

[18] Tang W, Chen Z, Zhang W, Cheng Y, Zhang B, Wu F, Wang Q, Wang S, Rong D, Reiter FP, et al. The mechanisms of sorafenib resistance in hepatocellular carcinoma: theoretical basis and therapeutic aspects. Signal Transduct Target Ther. 2020; 5(1):87. doi:10.1038/s41392-020-0187-x. 32532960 PMC7292831

[19] Li Q, Chen K, Zhang T, Jiang D, Chen L, Jiang J, Zhang C, Li S. 2023. Understanding sorafenib-induced ferroptosis and resistance mechanisms: implications for cancer therapy. Eur J Pharmacol. 955:175913. doi:10.1016/j.ejphar.2023.175913. 37460053

[20] Ratnadiwakara M, Anko ML. mRNA stability assay using transcription inhibition by actinomycin D in mouse pluripotent stem cells. Bio Protoc. 2018;8(21):e3072. doi:10.21769/BioProtoc.3072. PMC8342049 34532533

[21] Wenbin H, Kunling C, Yishi L, Donghui Z, Yuan C, Liuran L, Huang W, He G, Liao H, Cai L, et al. ABCC5 facilitates the acquired resistance of sorafenib through the inhibition of SLC7A11-induced ferroptosis in hepatocellular carcinoma. Neoplasia. 2021; 23(12):1227–1239. doi:10.1016/j.neo.2021.11.002. 34768109 PMC8591347

[22] Jia L, Ji Ling J, Yi Mei C, Wei Qi LJJPCR. KLF2 inhibits colorectal cancer progression and metastasis by inducing ferroptosis via the PI3K/AKT signaling pathway. J Pathol: Clin Res. 2023;9(5):423–435. doi:10.1002/cjp2.325. 37147883 PMC10397377

[23] Hua S, Chao P, Yang LJFCDB. Regulation of ferroptosis by PI3K/Akt signaling pathway: a promising therapeutic axis in cancer. 2024; 12. 10.3389/fcell.2024.1372330 PMC10982379 38562143

[24] Cohen PA, Jhingran A, Oaknin A, Denny L. Cervical cancer. Lancet. 2019;393(10167):169–182. doi:10.1016/S0140-6736(18)32470-X. 30638582

[25] Deng L-J, Deng W-Q, Fan S-R, Chen M-F, Qi M, Lyu W-Y, Qi Q, Tiwari AK, Chen J-X, Zhang D-M, et al. m6A modification: recent advances, anticancer targeted drug discovery and beyond. Mol Cancer. 2022; 21(1):52. doi:10.1186/s12943-022-01510-2. 35164788 PMC8842557

[26] Xie Q, Li Z, Luo X, Wang D, Zhou Y, Zhao J, Gao S, Yang Y, Fu W, Kong L, et al. piRNA-14633 promotes cervical cancer cell malignancy in a METTL14-dependent m6A RNA methylation manner. J Transl Med. 2022; 20(1):51. doi:10.1186/s12967-022-03257-2. 35093098 PMC8802215

[27] Tang D, Cao F, Yan C, Cui J, Guo X, Cheng L, Li L, Li Y-L, Ma J-M, Fang K, et al. Acinar cell-derived extracellular vesicle MiRNA-183-5p aggravates acute pancreatitis by promoting M1 macrophage polarization through downregulation of FoxO1. Front Immunol. 2022;13:869207. doi:10.3389/fimmu.2022.869207. 35911777 PMC9326086

[28] Plays M, Müller S, Rodriguez R. Chemistry and biology of ferritin. Metallomics. 2021;13(5). doi:10.1093/mtomcs/mfab021. PMC8083198 33881539

[29] Di Sanzo M, Quaresima B, Biamonte F, Palmieri C, Faniello MC. FTH1 pseudogenes in cancer and cell metabolism. Cells. 2020;9(12):2554. doi:10.3390/cells9122554. 33260500 PMC7760355

[30] Zhu M, Shen W, Wang Q, Zhou X, Wang J, Wang T, Zhang J. DARS-AS1 recruits METTL3/METTL14 to bind and enhance DARS mRNA m6A modification and translation for cytoprotective autophagy in cervical cancer. RNA Biol. 2022;19(1):751–763. doi:10.1080/15476286.2022.2079889. 35638109 PMC9176263

[31] Kong N, Chen X, Feng J, Duan T, Liu S, Sun X, Chen P, Pan T, Yan L, Jin T, et al. Baicalin induces ferroptosis in bladder cancer cells by downregulating FTH1. Acta Pharm Sin B. 2021; 11(12):4045–4054. doi:10.1016/j.apsb.2021.03.036. 35024325 PMC8727776

[32] Tian Y, Lu J, Hao X, Li H, Zhang G, Liu X, et al. FTH1 inhibits ferroptosis through ferritinophagy in the 6-OHDA model of Parkinson’s disease. Neurotherapeutics. 2020;17:1796–1812. doi:10.1007/s13311-020-00929-z. 32959272 PMC7851296

[33] Zhang R, Pan T, Xiang Y, Zhang M, Xie H, Liang Z, Chen B, Xu C, Wang J, Huang X, et al. Curcumenol triggered ferroptosis in lung cancer cells via lncRNA H19/miR-19b-3p/FTH1 axis. Bioact Mater. 2022;13:23–36. doi:10.1016/j.bioactmat.2021.11.013. 35224289 PMC8843976

[34] Hu Z-W, Wen Y-H, Ma R-Q, Chen L, Zeng X-L, Wen W-P, Sun W. Ferroptosis driver SOCS1 and suppressor FTH1 independently correlate with M1 and M2 macrophage infiltration in head and neck squamous cell carcinoma. Front Cell Dev Biol. 2021;9. doi:10.3389/fcell.2021.727762. PMC8437260 34527677

